# A Novel Approach for Oral Delivery of Insulin via *Desmodium gangeticum* Aqueous Root Extract

**DOI:** 10.4103/0975-1483.63158

**Published:** 2010

**Authors:** GA Kurian, AV Seetharaman, NR Subramanian, J Paddikkala

**Affiliations:** *School of Chemical and Biotechnology, SASTRA University, Thirumalaisamudram, Thanjavur, Tamil Nadu-613 402, India*; 1 *B.Tech (Biotechnology), SASTRA University, Amalanagar, Trichur, Kerala, India*; 2 *Department of Plant Biotechnology, Amala Cancer Research Center, Amalanagar, Trichur, Kerala, India*

**Keywords:** *Desmodium gangeticum*, glucose tolerance test, oral insulin, streptozotocin-induced diabetes

## Abstract

Many challenges are associated with the oral delivery of insulin, relating to the physical and chemical stability of the hormone, and its absorption and metabolism in the human body. The present study aims to demonstrate the oral delivery of insulin in both normal and steptozotocin (STZ)-induced diabetic rats with the help of the aqueous extract of *Desmodium gangeticum* (DG) root. Human insulin was mixed with the aqueous extract of DG root (0.1 mg/ml) with human insulin (40 IU/ml) in ratio 1:1(v/v), to prepare oral insulin drug. Decreased plasma glucose level and increased plasma insulin in normal and STZ-induced diabetic rat suggested the probable absorption of insulin through GI tract when insulin was administered by mixing with DG extract. Indeed, insulin mixed DG potentially stimulates the release of insulin in STZ-induced diabetic rat rather than in normal animal. *In vivo* insulin secretaguage action of oral insulin drug was determined by isolated rat heart model and the results showed a significant cardio protection in STZ rat. The finding of this study suggests that insulin mixed with DG extract can be a promising vehicle for oral delivery of insulin. However, further studies are required to explore the exact compound(s) responsible for the protective delivery of insulin orally. Increased plasma insulin level by insulin mixed DG extract administration in STZ-treated diabetic rat indicates not only insulin secretaguage action of the mixture but also a probable altered insulin release mechanism in diabetic condition.

## INTRODUCTION

Insulin is a natural hormone which controls the level of blood glucose and are limited in tissues of diabetes mellitus (DM)-induced rats. Patients with Type 2 DM are insulin resistant, have relatively low insulin production, or both. Some patients with Type 2 diabetes may eventually require insulin when other medications fail to control blood glucose levels adequately.

Many new pharmacological agents have been added to our armamentarium of treatments for DM in the last decade. The goal of all treatments is the same irrespective of the cause of the DM, namely to normalize blood glucose. Insulin therapy affords effective glycemic control, yet its short comings such as ineffectiveness on oral administration, short shelf life, and requirement of constant refrigeration.[1] Treatment with sulfonylureas and biguanides is also associated with side effects.[2] However, recently numerous researches are being carried out to reduce the limitations of insulin therapy.

Researchers are trying to find various alternatives to deliver insulin via noninvasive routes, such as nasal,[3] rectal,[4] pulmonary,[5] and ocular deliveries.[6] However, among all alternative routes for the administration of insulin, the oral route is the most convenient. In addition, because orally administered insulin undergoes a first hepatic pass, it will produce a similar effect as pancreas-secreted insulin by inhibiting the hepatic gluconeogenesis and suppressing the hepatic glucose production.[7] Unfortunately, oral delivery of peptides or proteins such as insulin poses unique problems of instability, susceptibility to proteolysis, and inability to traverse membranes and biological barriers due to their large molecular size.[8] As a result, the absolute amount of intact protein reaching the target site is too small to be of pharmacological benefit. To overcome these major problems, it was suggested to increase the oral absorption of insulin to circumvent the digestion of this polypeptide in the gastrointestinal (GI) tract by entrapping insulin in polymeric microspheres[9] or by coating with polymer films.[10] However, no study has been done to transport insulin with the help of an herbal extract as a vehicle.

*Desmodium gangeticum* (DG) (Leguminosae) is a shrub common on the lower hills and plains throughout India. DG is widely used in the indigenous system of medicine in India and is reported to contain flavone and isoflavonoid glycoside.[11] It forms the ingredient of many Ayurvedic formulations used for diabetes. Moreover, it strengthens the nervous system, improves digestion, and secretion of digestive enzymes.[11] It also acts as diuretics. In this study we used insulin mixed with an extract (aqueous) obtained from DG root as a vehicle to transport insulin across the GI tract.

## MATERIALS AND METHODS

Preparation of aqueous extract of the roots of *Desmodium gangeticum*

The plant after collection from the herbal garden maintained in the department was washed and cleaned. The plant material was taxonomically identified and the voucher specimen A/C no. 3908 was retained in our laboratory for future reference.

One kilogram (1 kg) of fresh secondary roots of DG were sliced and air-dried at room temperature. The sliced, air-dried roots of the plant were milled into fine powder. Soxhlet extraction with 2.5 l of distilled water at room temperature for 24 h with shaking was used to obtain the extracts. The aqueous extracts were filtered and concentrated to dryness under reduced pressure at 30±1°C. The resulting aqueous extract was freeze-dried, finally giving 18.66 g [i. e., 1.866% yield]. Aliquot portions of the crude root aqueous extract residue were weighed and dissolved in distilled water for the experiment.

### Animals

Male wistar rats (170-190 g) were procured from King Institute of Preventive Medicine, Chennai, India, and used for the investigation of oral delivery of insulin via DG. These studies were approved by the Institutional animal ethical committee (IAEC). The animals were housed under standard conditions of temperature (22±3°C) and relative humidity (30-70%) with a 12:12 light: dark cycle. The animals were fed with standard pellet diet (Amrit Feeds Ltd, Bangalore.) and water ad libitum. Animals were fasted for 18-24 h and streptozotocin (STZ; 65 mg/kg) in 0.02 M citrate saline buffer was administered intraperitoneally, as described previously.[12] A blood glucose level exceeding 200 mg/dl was considered diabetic.

### Preparation of DG-mixed insulin

DG-mixed insulin mixtures were prepared by mixing the aqueous extract of DG (1 mg/ml) with human insulin (40 IU/ml) in ratio 1:1(v/v).

### Oral glucose tolerance test

Oral glucose tolerance test was performed in normal and diabetic rats after an overnight fasted (18 h). Animals were randomized into following groups: (1) normal control; (ii) diabetic control; and (iii)drug group. In fact drug groups were again divided into two subgroups: (a) effect of drug on OGTT in normal rat and (b) effect of drug on OGTT in diabetic rats. Animals were fasted overnight and were placed in restraining cages for 30 min in the morning before commencing the study. Glucose was given orally by an esophageal tube as a 40% solution (1 g/kg body wt). The drugs were administered both intraperitoneal and oral routes. Blood was withdrawn from the orbital plexus of each animal at 0, 30, 60, 90,120, and 180 min after administration of the oral load. The fasting blood glucose levels were estimated by using standard kits from Qualigens India. Plasma insulin was analyzed by the IRMA method.

Isolated heart preparation and ischemia-reperfusion experiment for insulin secretogenic action

Rats were heparinized (500 IU/kg, i.p.) and anesthetized with pentobarbital (50 mg/kg, i.p.), and the heart was isolated and perfused retrogradely using the Langendorff method with Krebs-Henseleit buffer (pH 7.4, in mM) 118 NaCl, 4.7 KCl, 2.5 CaCl_2_, 1.2 MgSO_4_, 1.2 KH_2_PO_4_, 25.0 Na_2_HCO_3_, 11.0 glucose,) equilibrated with 95% O_2_and 5% CO_2_ gas mixture at 36.5°C at a constant pressure of 80 mmHg. The coronary perfusion pressure was maintained at 80 mmHg. The left ventricular pressure developed within ventricle filled with Krebs solution was recorded with a pressure transducer, which in turn was connected to a device amplifier and chart recorder. This left ventricular pressure gave an indication of the mechanical performance of the heart. Coronary flow was measured simply by collecting the perfusate draining from the heart in a graduated cylinder for a defined time. The heart rate was measured by counting the number of contractions (obtained from the left ventricular pressure record) per minute.

Group A: Normal rat hearts (n=6) subjected to 70 min perfusion with KH buffer.

Group B: Normal rat hearts (n=6) subjected to 20 min global ischemia followed by 30 minutes reperfusion after 20 min equilibration.

Group C: Diabetic rat hearts (n=6) subjected to 20 min global ischemia followed by 30 min reperfusion after 20 min equilibration.

Group D: Normal rat hearts (n=6) were pretreated with oral insulin before 1 h of experiment and then subjected to IR injury similar to group B.

Group E: Diabetic rat hearts (n=6) were pretreated with oral insulin before 1 h of experiment and then subjected to IR injury similar to group C.

Group F: Normal rat hearts (n=6) were subjected to 20 min global ischemia and oral insulin was given along with KH buffer prior to 30 min reperfusion.

Group G: Diabetic rat hearts (n=6) were subjected to 20 min global ischemia and oral insulin was given along with KH buffer prior to 30 min reperfusion.

The coronary effluent during the 30 min of reperfusion period was collected for measurement of creatine kinase content (released CK) and lactate dehydrogenase. Lactate dehydrogenase (LDH) and creatine phosphokinase (CPK) were determined by the method of King[13] and Okinaka and his coworker,[14] respectively, using commercially available kit. Blood glucose was measured by the glucose-oxidase method.[15] Insulin was assayed by the radio-immunoassay kit method.

### Statistical analysis

All data were reported as mean ± SD. Results were statistically analyzed by a one-way analysis of variance (ANOVA) by SPSS software 12.00, followed by Duncan’s multiple range test (DMRT). P> 0.05 was considered to be significant.

## RESULTS

### Oral glucose tolerance test

**Figure 1:**
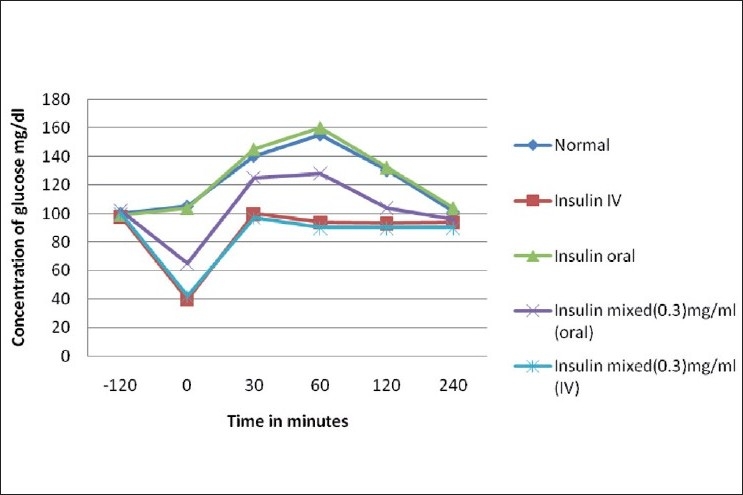
Glucose tolerance test in normal rats

**Figure 2:**
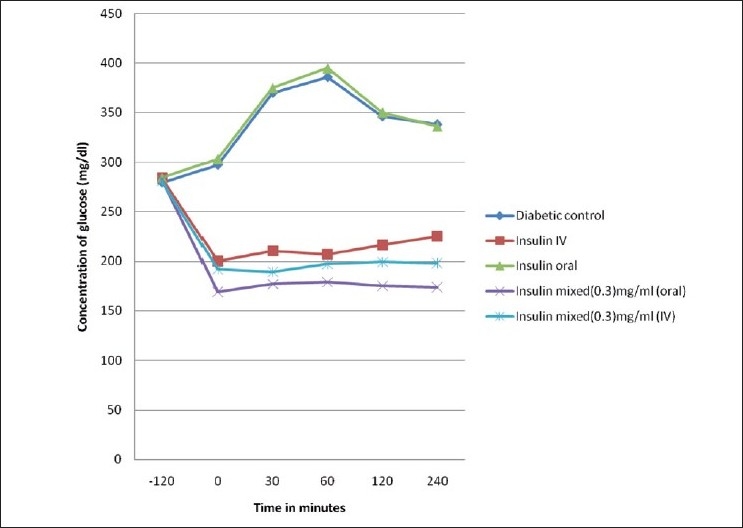
Glucose tolerance test in diabetic rats

The mean glucose response is presented in Figures 1 and 2. In oral glucose tolerance test, the blood glucose level increased after glucose administration and reached a peak concentration at 60 min. Around 120 min after glucose administration, the blood glucose level decreased in normal and slightly declined in diabetic control rats. On comparison with intraperitoneal administered insulin, orally administrated insulin in the OGTT procedure did not reduce glucose concentration after 60 min. However, oral administration of DG-mixed insulin showed a significant decline in glucose level after 90 min. In fact, insulin mixed with 3 mg/ml of DG extract showed better result. In contrast, 1 h prior administration of drugs did not show any significant impact on the changes in pattern of glucose concentration.

### Insulin and glucose level

Table 1 shows plasma insulin and glucose levels in normal and diabetic rats. In normal rats, plasma insulin concentrations were elevated when the insulin was given intraperitoneal and as insulin mixed with DG. A subsequent decline in blood glucose level confirms the activity of insulin delivered in both routes (intraperitoneal and oral). In fact, oral administration of insulin did not show any significant elevation of plasma insulin levels.

**Table 1:** Concentration of plasma insulin and plasma glucose in normal and diabetic rats treated with insulin and insulin-mixed DG

Groups	Plasma insulin (Units/ml plasma)	Plasma glucose (mmol/l)
Normal rats		
Normal	^a^74± 8.3	^a^2.46± 0.2
Normal rats given insulin intraperitoneal route	^d^102± 12.1*	^d^1.23± 0.7*
Normal rats given insulin oral route	^a^72± 8.3	^a^2.48± 0.5
Normal rats given aq. extract of DG(0.3 mg/kg b.wt) intraperitoneal route	^a^74± 6.7	^a^2.46± 0.3
Normal rats given aq. extract of DG(0.3 mg/kg b.wt) oral route	^a^75± 6.2	^a^2.42± 0.4
Normal rats given insulin-mixed DG (0.3 mg/kg b.wt) intraperitoneal route	^d^96± 8.6*	^d^1.36± 0.2*
Normal rats given insulin-mixed DG(0.3 mg/kg b.wt) oral route	^d^90± 7.4*	^d^1.45± 0.5*
STZ-induced diabetic rats		
Diabetic control	^b^50± 6.2	^b^7.89± 0.5
Diabetic rats given insulin oral	^b^52± 4.7	^b^7.69± 0.9
Diabetic rats given insulin intraperitoneal	^a^70± 6.3*	^a^2.43± 0.9*
Diabetic rats given aq. extract of DG(0.3 mg/kg b.wt) intraperitoneal	^b^57± 5.1	^b^7.52± 0.6
Diabetic rats given aq. extract of DG(0.3 mg/kg b.wt) oral	b59± 5.3	b7.47± 0.5
Diabetic rats given insulin-mixed DG(0.3 mg/kg b.wt) intraperitoneal	^c^80± 7.3*	^c^2.29± 0.2*
Diabetic rats given insulin-mixed DG(0.3 mg/kg b.wt) oral	^d^97± 7.3*	^d^1.60± 0.3*

Values are mean ± SD for six rats in each group

Significantly differ from normal control group (normal rats are compared with normal control and STZ induced rats with diabetic control) are expressed as (*) *P*< 0.01

Values not sharing a common superscript (a,b,c,d) differ significantly at *P*<0.05 when compared between the groups.

A similar pattern of results were observed in diabetic rats also. But, to our surprise the elevation of plasma insulin levels were much higher than that of normal animals when insulin-mixed DG was used as the vehicle to transport insulin. We predict that the above results may be due to insulin secretagouge action of DG-mixed insulin.

### Isolated heart preparation and ischemia-reperfusion experiment

**Figure 3:**
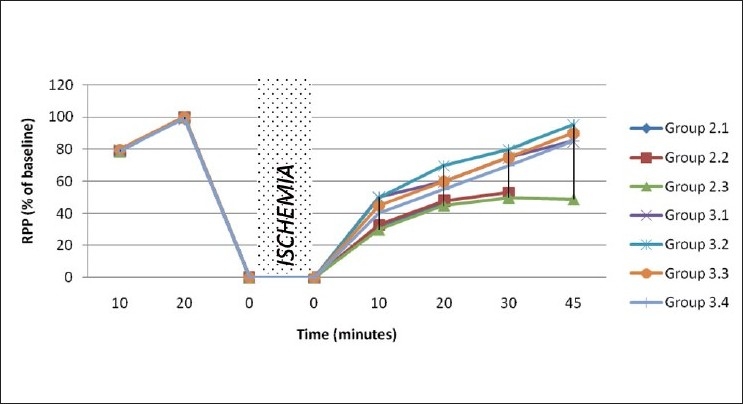
Rate pressure product of hearts subjected to 30 minutes global ischemia followed by reperfusion in normal rats

**Figure 4:**
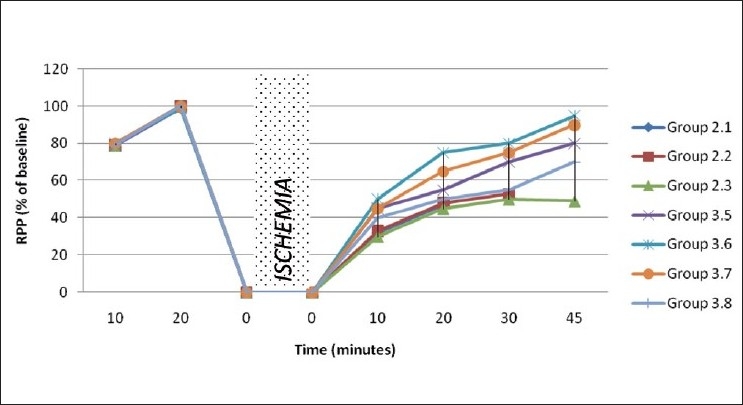
Rate pressure product of hearts subjected to 30 minutes global ischemia followed by reperfusion in diabetes mellitus rats

Absolute values of hemodynamic parameters were shown in Figures 3 and 4. Depressed physiological parameters in ischemia reperfusion control were recovered in rats that received DG-mixed insulin through both intraperitoneal and oral route. Interestingly, diabetic rats showed a better preservation of hemodynamic parameters like LVDP, heart rate and RPP, when they were given DG-mixed insulin (both intraperitoneal and oral route administered drug) than normal rats.

Table 2 and 3 show the amount of released CK and LDH during reperfusion stage of the experiment. When the heart was perfused with DG-mixed insulin, amount of released CK and LDH was found to be significantly lowered than normal ischemia reperfusion control. But the functional recovery of myocardium by DG-mixed insulin in terms of released LDH and CK was more significant in diabetic animal than normal animal. Cardiac enzymes in the tissue homogenate also complimented the above results.

**Table 2:** Activity of CK, LDH, SGOT, and SGPT in the tissue homogenate of isolated rat heart

Group	CK (μmol phosphorous generated/min/mg protein)	LDH (nmol pyruvate generated /min/mg protein)	SGOT (nmol pyruvate generated /min/mg protein)	SGPT (nmol pyruvate generated /min/mg protein)
A	a17.3± 1.3	a106.4 ± 7.2	a38.2 ± 2.1	a24.6± 1.6
B	b8.7± 0.3	b60.1± 4.2	b18.2 ± 1.2	b15.5± 1.1
C	b6.33±0.9	d40.6± 4.2	b19.67± 1.1	b14.23± 1.3
D	c12.11± 2.1	b72.6± 8.1	b18.2± 1.3	b17.3± 2.1
E	c10.52± 1.3	b52.6± 4.2	b20.3 ± 3.2	b16.6± 1.8
F	c13.42± 1.3	c87.3±4.3	a32.2± 1.3	a21.2± 1.4
G	a15.46± 1.4	c88.6± 5.4	a32.4± 2.4	a23.3± 1.1

Values are mean ± SD for six rats in each group

Values not sharing a common superscript (a,b,c,d) differ significantly at *P*<0.05) when compared between the groups

**Table 3:** Activity of creatine kinase and lactate dehydrogenase in the perfusate of isolated rat heart

Group	CK (Units/ml/perfusate)	LDH (Units/ml/perfusate)
A	^a^175± 23.2	^a^70.1± 10.2
B	^b^320± 43.3	^b^145.2± 20.4
C	^b^330± 45.8	^b^155.6± 23.6
D	^c^281± 22.6	^c^102.2± 14.9
E	^c^290± 23.7	^c^125.5± 18.3
F	^d^194± 22.8	^c^101.2± 20.8
G	^a^172± 16.1	^a^87.5± 14.1

Values are mean ± SD for six rats in each group

Values not sharing a common superscript (a,b,c,d) differ significantly at *P*<0.05) when compared between the groups

## DISCUSSION

The important findings of this study can be summarized as follows: (i) when aqueous extract of DG root was used as a vehicle to transport insulin, it not only helps insulin to get absorbed across the GI tract but also in active form; (ii) a significant increase in plasma insulin in diabetic rats as compared to normal rats when insulin was given along with its vehicle predicted a probable insulin secretagouge action of DG-mixed insulin and (iii) oral administration of insulin along with DG aqueous extract showed significant cardio protection to ischemia reperfusion injury in rats.

### Oral Glucose tolerance test

Steptozotocin-induced diabetic rats presented significant hyperglycemia as compared to normal rats in OGTT analysis. Intraperitoneal administration of insulin, insulin along its vehicle, and oral administration of insulin-mixed DG normalizes the blood glucose level after 90/120 min [Figures 1 and 2]. These findings are in agreement with the literature that indicates that insulin normalizes the metabolic control of diabetic rats.[16] However, significant hypoglycemic action mediated by insulin-mixed DG administered by both intraperitoneal and oral route predicted either increased insulin level or increased half life of insulin in blood.

### Plasma insulin glucose level

The hypoglycemic effect was taken as a monitor for insulin absorption in its physiologically active form. The results are shown in Table 1. When insulin-mixed DG was given, a significant reduction in plasma glucose along with increased plasma insulin in both normal and diabetic rats was observed, indicating a direct response of insulin. In normal rats, OGTT showed a prominent hypoglycemic effect at 30 to 90 min and 60 to 120 min in insulin-mixed DG rats given intraperitoneal and oral route, respectively, and the effect was visible up to 180 min [Figure 1]. The maximum plasma glucose reduction (% of initial) was found to be 78% ± 2% and the time to reach the maximum plasma glucose reduction was 1.30 and 2 h in intraperitoneal administered rats and oral administered rats, respectively. In fact, orally administered insulin at the same dose showed no reduction in blood glucose which indicates proteolytic degradation of insulin in the GI tract or presystemic metabolism. These results suggested that DG extract can not only protect insulin against *in vivo* proteolytic degradation but can also preserve the biological activity of insulin. Numerous works have reported that on hypoglycemic activity of insulin loaded with nanosphere,[17] microsphere,[18] and other vehicle, none of the interventions were effective as compared to insulin administered directly to blood.

However, in diabetic rats, hypoglycemic effect mediated by DG-mixed insulin was more significant as compared to normal rats [Table 1]. It is worth to mention here that DG extract can release insulin from Islet cells of Langerhans.[19] Hence, the prominent level of plasma insulin in diabetic rats and the subsequent decline in plasma glucose level indicate insulin secretagogue action of DG extract. However, the reason for significant insulin secretagogue action of DG-mixed insulin in diabetic rat is highly debatable as we could not find similar action in normal rats. Moreover, administration of DG extract alone was unable to increase the plasma insulin level significantly. From the above observation, we suspect a change in metabolic release of insulin in diabetic rats, which needs to be proved further.

### Isolated heart preparation and ischemia-reperfusion experiment

*In vivo* physiological activity of insulin in DG-mixed insulin was assessed in an experimental model for ischemia reperfusion injury (IRI)-induced diabetic rat heart. Hyperglycemic conditions significantly exacerbate the anomalies associated with IRI[20] and block the cardio-protective effect mediated by glucose insulin potassium. Functional recovery of rat heart treated with DG-mixed insulin measured through hemodynamic parameters suggested myocardial protection.

The decreased activities of CPK, LDH, AST, and ALT in the myocardium and corresponding increase of cardiac markers in the perfusate are one of the indicators for cardiac injury associated with ischemia reperfusion, shown in ischemia reperfusion control rats. Oral and intraperitoneal administration of DG-mixed insulin significantly reduced the CK and LDH levels in perfusate, indicating the preservation of myocardial architecture during ischemia reperfusion.

## CONCLUSION

The present study showed an innovative approach to administered insulin through oral route. In fact this study also provide clue for the difference in the insulin secretion mechanism in normal and diabetic rats. However, further studies need to be carried out to find the exact compound and mechanism involved in the above findings.
